# Short versus long duration of ceftaroline combination therapy and outcomes in persistent or high-grade MRSA bacteremia: A retrospective single-center study

**DOI:** 10.1371/journal.pone.0304103

**Published:** 2024-05-20

**Authors:** M. Gabriela Cabanilla, Michael L. Bernauer, Liana M. Atallah, Matthew J. Briski, Jason Koury, Cecilia M. Thompson, Chelsea N. Rodriguez, Bernadette Jakeman, Thomas F. Byrd

**Affiliations:** 1 Division of Infectious Diseases, Department of Internal Medicine, University of New Mexico Health Sciences Center, Albuquerque, New Mexico, United States of America; 2 Department of Pharmacy, University of New Mexico Health Sciences Center, Albuquerque, New Mexico, United States of America; 3 RS21, Albuquerque, New Mexico, United States of America; 4 TriCore Reference Laboratories, Albuquerque, New Mexico, United States of America; 5 Department of Pharmacy Practice and Administrative Sciences, University of New Mexico College of Pharmacy, Albuquerque, New Mexico, United States of America; Aga Khan University, PAKISTAN

## Abstract

**Background:**

Methicillin-resistant *Staphylococcus aureus* (MRSA) is associated with high mortality rates. Despite antibiotic therapy, persistent bacteremia is challenging to treat. Combination therapy with ceftaroline has emerged as a potential treatment option; however, the optimal duration and clinical implications after bacteremia clearance are unknown.

**Methods:**

This retrospective cohort study examined patients with high-grade or persistent MRSA bacteremia who were treated with ceftaroline combination therapy at the University of New Mexico Hospital between January 2014 and June 2021. Patients were categorized into short- (<7 days) or long-duration (≥7 days) groups based on the duration of combination therapy after bacteremia clearance. Outcomes included 30-day all-cause mortality, bacteremia recurrence, post-bacteremia clearance length of stay, and adverse events.

**Results:**

A total of 32 patients were included in this study. The most common sources of bacteremia were bone/joint and endovascular (28.1%, 9/32 each). The median duration of combination therapy after clearance was seven days (IQR 2.8, 11). Patients in the long-duration group had a lower Charlson comorbidity index (1.0 vs 5.5, p = 0.017) than those in the short-duration group. After adjusting for confounders, there was no significant difference in the 30-day all-cause mortality between the groups (AOR 0.17, 95% CI 0.007–1.85, p = 0.18). No association was found between combination therapy duration and recurrence (OR 2.53, 95% CI 0.19–inf, p = 0.24) or adverse drug events (OR 3.46, 95% CI 0.39–74.86, p = 0.31). After controlling for total hospital length of stay, there was no significant difference in the post-bacteremia clearance length of stay between the two groups (p = 0.37).

**Conclusions:**

Prolonging ceftaroline combination therapy after bacteremia clearance did not significantly improve outcomes in patients with persistent or high-grade MRSA bacteremia. The limitations of this study warrant cautious interpretation of its results. Larger studies are needed to determine the optimal duration and role of combination therapy for this difficult-to-treat infection.

## Introduction

*Staphylococcus aureus* is a pathogen of clinical interest because of its high mortality rate in bacteremia of up to 30% [1]. Specifically, methicillin-resistant *Staphylococcus aureus* (MRSA) bacteremia has been associated with a large clinical burden, increased mortality, and high rates of treatment failure with first-line treatment options [2–4]. According to a 2020 United States Centers for Disease Control and Prevention (CDC) report, it caused over 70,000 infections nationwide, with approximately 9,000 deaths annually [5].

Persistent bacteremia has been previously defined as the continuation of positive blood cultures for seven days or more, despite adequate antibiotic therapy [6, 7]. However, a recent study by Kuehl et al. suggested redefining the cutoff duration for persistent bacteremia as two days or more of blood culture positivity [8]. Much controversy surrounds the definition of persistent bacteremia and the optimal treatment approach, leading to a lack of consensus among healthcare providers. What remains undisputed is that persistent bacteremia is associated with an increased incidence of metastatic sites of infection, poor patient outcomes, and increased healthcare costs [1]. Despite efforts to achieve source control, persistent bacteremia may occur, leading to treatment failure even in patients receiving optimal antibiotic therapy [7].

The Infectious Diseases Society of America (IDSA) guidelines recommend vancomycin monotherapy as the first-line agent with patient assessment to determine if a change in therapy is appropriate around day seven of persistent bacteremia [7]. However, the guidelines are not clear on recommendations for an alternative or salvage regimen, and do not state a preference for any agent. Additionally, there is a growing body of evidence for combination therapies for initial treatment or salvage therapy in the setting of persistent MRSA bacteremia.

Ceftaroline has emerged as a potential salvage therapy option because it is an anti-staphylococcal beta-lactam with added benefits in terms of its activity against MRSA. Prior evidence has shown that the combination of vancomycin or daptomycin with ceftaroline may lead to a shorter duration of MRSA bacteremia [9]. However, the indications for its use, optimal combination, dose, timing, and impact on clinical outcomes remain unclear. There is also no consensus on the treatment duration for salvage or combination therapy after bacteremia clearance. We aimed to examine the effect of ceftaroline combination therapy duration following the clearance of persistent or high-grade MRSA bacteremia on various clinical outcomes (30-day mortality, bacteremia recurrence, post-bacteremia clearance length of stay, and adverse drug events [ADE]).

## Materials and methods

### Study design and setting

This single-center, retrospective cohort study examined patients with high-grade or persistent MRSA bacteremia who received ceftaroline combination therapy while hospitalized at the University of New Mexico Hospital (UNMH). UNMH is a publicly funded tertiary care center located in Albuquerque, New Mexico, serving as the primary safety net hospital for the state, providing care to a largely underserved population [10]. Patients were categorized into two groups. Those who received combination therapy with ceftaroline for less than seven days after bacteremia clearance were assigned to the short-duration group. We assigned those who received seven days or more of combination therapy after bacteremia clearance to the long-duration group.

### Study population and data collection

Patients were included if they were 18 years or older, admitted to UNMH from January 1, 2014 to June 30, 2021, had high-grade or persistent MRSA bacteremia, and received combination therapy with ceftaroline plus vancomycin or daptomycin. Patients were excluded if they did not meet the inclusion criteria, had polymicrobial bacteremia, were incarcerated, or did not receive initial monotherapy with either vancomycin or daptomycin at the time of a positive blood culture. The study period was selected based on the availability of ceftaroline at our institution.

The decision to add ceftaroline as combination therapy with either vancomycin or daptomycin was at the discretion of the treating physician, as there is no current local, national, or international guidance on this practice. Ceftaroline is a restricted medication at our institution and requires infectious diseases consultation for approval. Relevant patient demographics and clinical and treatment data were extracted from electronic medical records and entered into a secure electronic data form by the study investigators.

### Study outcomes and definitions

The primary outcome measure was 30-day all-cause mortality. Secondary outcomes included bacteremia recurrence, post-bacteremia clearance length of stay, and adverse drug events (i.e., acute kidney injury [AKI], neutropenia, and *C*. *difficile* colitis).

High-grade MRSA bacteremia, as observed in our local practice, was defined as evidence of MRSA growth in two or more sets of blood cultures on a single day. Persistent bacteremia, as per recently proposed definitions in the literature [8, 11], was defined as evidence of MRSA growth in at least one set of blood cultures on two or more consecutive days. Bacteremia clearance was defined as at least two negative blood cultures in a single day or two negative blood cultures taken on consecutive days if only one set was drawn per day. Source control was defined as intravenous catheter removal, drainage, other debridement (including amputation), valve replacement, or chest tube placement based on the source of infection.

AKI was defined as an increase in serum creatinine of ≥ 0.3 mg/dL within 48 hours of anti-MRSA antibiotic start or ≥ 1.5 times from baseline. We defined neutropenia as an absolute neutrophil count of < 1500 cells/mm^3^ during antibiotic therapy. *C*. *difficile* colitis was defined as a positive *C*. *difficile* polymerase chain reaction after antibiotic therapy initiation and up to 30 days after antibiotic discontinuation. Bacteremia recurrence was defined as at least one set of positive blood cultures for MRSA after documented bacteremia clearance and up to 6 months after the initial treatment completion. Post-bacteremia clearance length of stay was defined as the length of hospital stay in days after bacteremia clearance.

### Statistical analysis

Descriptive summaries of continuous variables were reported using medians and interquartile ranges. Univariate analysis of categorical variables was done using *χ*2 and Fisher’s exact tests. Yate’s continuity correction was used for sparse variables. Univariate analysis of continuous variables was performed using two-sample t-tests for normally distributed variables, and Wilcoxon rank-sum tests for non-normally distributed variables. We assessed normality through visual inspection and Shapiro-Wilk tests.

Multiple logistic regression was used to test associations between the treatment variable (duration of combination therapy after bacteremia clearance ≥ 7 days) and binary outcome variables (e.g., 30-day mortality, bacteremia recurrence, and any ADE), while multiple linear regression was used for continuous outcomes (i.e., post-bacteremia clearance length of stay). In addition to multiple linear regression, a univariate test of post-bacteremia clearance length of stay was conducted between patients receiving short-duration combination therapy (< 7 days) and long-duration combination therapy (≥ 7 days) using Wilcoxon rank-sum tests.

For multiple logistic regression and multiple linear regression, the main effect terms included the treatment variable and covariates that were significantly associated with the outcome in the univariate analysis. Additional terms were included in the logistic regression model if they demonstrated indications of modifying the treatment variable or other factors, typically characterized by a change in the coefficient by at least 15% (i.e., %*Δβ*≥15). Statistical significance was set at a P value of ≤0.05, and we completed the analysis using R, version 4.2.3 (R Foundation for Statistical Computing, Vienna, Austria).

### Ethics

The study was approved by the Human Research Review Committee of the University of New Mexico Health Sciences Center (protocol #21–329). Owing to the retrospective nature of this study, the need for informed consent was waived by the ethics committee. Data were accessed from July 1st to October 31st, 2022. Only the authors authorized by the Human Research Review Committee of the University of New Mexico Health Sciences Center had access to information that could identify individual participants during data collection.

## Results

**Fig 1:**
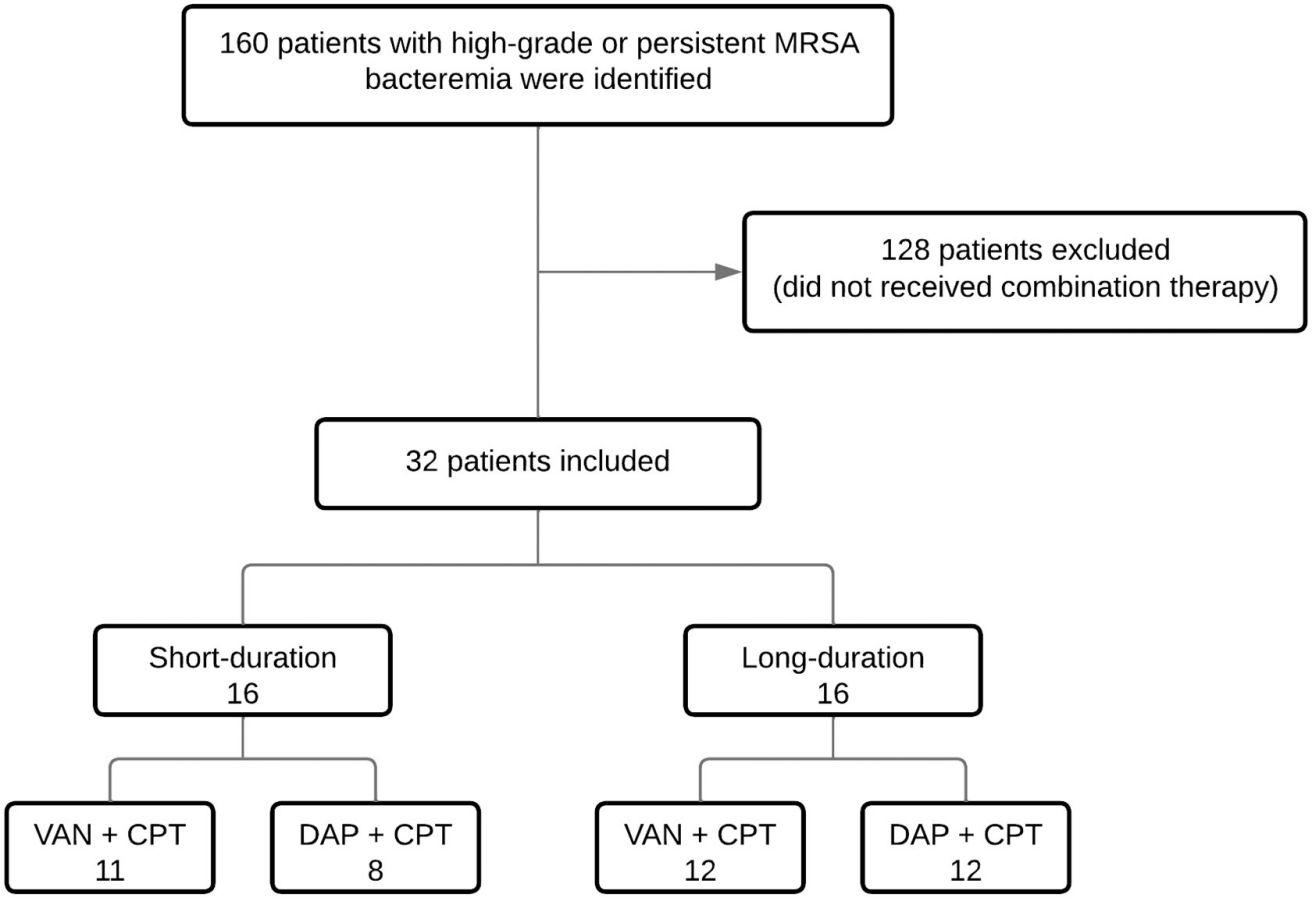
Study population. Short-duration refers to patients who received combination therapy for < 7 days. Long-duration refers to patients who received combination therapy for ≥ 7 days. Some patients received both VAN + CPT and DAP + CPT during their treatment course. Abbreviations: CPT, ceftaroline; DAP, daptomycin; MRSA, methicillin-resistant *Staphylococcus aureus*; VAN, vancomycin.

We initially identified 160 patients with high-grade or persistent MRSA bacteremia during the study period. Of the 160 patients, 128 were excluded (Fig 1). Thirty-two patients received combination therapy with ceftaroline. The median age of the patients in the cohort was 46.2 years (IQR 40.6, 64.1). Most patients were white (71.9%, 23/32) and male (59.4%, 19/32). Fifty percent of the patients (16/32) identified themselves as Hispanic or Latino. Most patients (59.4%, 19/32) had known substance use disorders (alcohol, tobacco, and/or illicit drugs, with or without injection drug use).

The majority of patients (96.9%, 31/32) met the criteria for high-grade MRSA bacteremia, and 22 patients (68.8%) had persistent bacteremia. The most common sources of bacteremia were bone and joint (28.1%, 9/32), endovascular (28.1%, 9/32), and respiratory (21.9%, 7/32) infections. The median Charlson comorbidity index (CCI) and Pitt bacteremia score (PBS) were 2.0 (IQR 0, 6) and 1.0 (IQR 0, 2.5), respectively. Table 1 presents the baseline characteristics of the study cohort.

**Table 1:** Baseline characteristics of patients treated with vancomycin or daptomycin in combination with ceftaroline.

	Total (n = 32)	Short-duration^a^ (n = 16)	Long-duration^b^ (n = 16)	P value
**Male**, n (%)	19 (59.4)	8 (50.0)	11 (68.8)	0.472
**Age**, median (IQR)	46.2 (40.6, 64.1)	55.6 (45.1, 73.5)	44.9 (40.1, 50.6)	0.060
**Race**, n (%)				0.970
White	23 (71.9)	12 (75.0)	11 (68.8)	
AI/AN	5 (15.6)	2 (12.5)	3 (18.8)	
Black/African American	2 (6.2)	1 (6.2)	1 (6.2)	
Unknown	2 (6.2)	1 (6.2)	1 (6.2)	
**Hispanic/Latino ethnicity**, n (%)	16 (50.0)	7 (43.8)	9 (56.2)	0.724
**Charlson comorbidity index**, median (IQR)	2.0 (0, 6)	5.5 (1, 6.5)	1.0 (0, 2.3)	0.017
**Pitt bacteremia score**, median (IQR)	1.0 (0, 2.5)	1.5 (0, 4)	1.0 (0.8, 2)	0.549
**Comorbidities**, n (%)				
Diabetes mellitus	13 (40.6)	8 (50.0)	5 (31.2)	0.472
Substance use disorder	19 (59.4)	9 (56.2)	10 (62.5)	1.000
Liver disease	7 (21.9)	4 (25.0)	3 (18.8)	1.000
Cardiovascular disease	7 (21.9)	4 (25.0)	3 (18.8)	1.000
Moderate-severe CKD	6 (18.8)	3 (18.8)	3 (18.8)	1.000
**Bacteremia source**, n (%)				0.208
Bone/joint	9 (28.1)	5 (31.2)	4 (25.0)	
Endovascular	9 (28.1)	2 (12.5)	7 (43.8)	
Respiratory	7 (21.9)	5 (31.2)	2 (12.5)	
SSTI	4 (12.5)	2 (12.5)	2 (12.5)	
Intravenous line	2 (6.2)	2 (12.5)	0 (0.0)	
**Infectious diseases consultation**, n (%)	32 (100)	16 (100)	16 (100)	–
**Endocarditis**, n (%)	15 (46.9)	6 (37.5)	9 (56.2)	0.479
**Source control obtained**, n (%)	14 (43.8)	5 (31.2)	9 (56.2)	0.285
**High-grade bacteremia**, n (%)	31 (96.9)	15 (93.8)	16 (100)	1.000
**Persistent bacteremia**, n (%)	22 (68.8)	10 (62.5)	12 (75.0)	0.703
**Received VAN + CPT**, n (%)	23 (71.9)	11 (68.8)	12 (75.0)	1.000
**Received DAP + CPT**, n (%)	20 (62.5)	8 (50.0)	12 (75.0)	0.273
**Time to combination therapy in days**, median (IQR)	6.0 (5, 8)	5.0 (4, 8.3)	6.0 (6, 8)	0.147
**Time from first antibiotic to bacteremia clearance in days,** median (IQR)	8 (6, 10.8)	8 (5.5, 10)	8.5 (6, 11.3)	0.739
**Combination duration after bacteremia clearance in days**, median (IQR)	7.0 (2.8, 11.0)	2.5 (0.8, 3.5)	11.0 (8.8, 13)	<0.001

Some patients received both VAN + CPT and DAP + CPT during their treatment course.

^a^Short-duration refers to patients who received combination therapy for < 7 days.

^b^Long-duration refers to patients who received combination therapy for ≥ 7 days.

Abbreviations: AI/AN, American Indian/Alaska Native; CKD, chronic kidney disease; CPT, ceftaroline; DAP, daptomycin; IQR, interquartile range; SSTI, skin and soft tissue infection; VAN, vancomycin.

The MRSA blood isolates in this cohort had a median vancomycin minimum inhibitory concentration (MIC) of 1 mg/L (IQR 1, 1) and daptomycin MIC of 0.5 mg/L (IQR 0.5, 0.5); ceftaroline susceptibilities were not routinely reported. Vancomycin was predominantly prescribed as initial monotherapy in this cohort (96.9%, 31/32). Most of the patients (71.9%, 23/32) received ceftaroline in combination with vancomycin. Combination therapy was initiated after a mean of 6.3 days from the initial blood culture with MRSA growth. The median total time from the first antibiotic to bacteremia clearance was 8 days (IQR 5.5, 10) in the short-duration group and 8.5 days (IQR 6, 11.3) in the long-duration group without a significant difference (p = 0.739). The median total duration of combination therapy after bacteremia clearance was 7 days (IQR 2.75, 11). The baseline characteristics were similar between the two groups (Table 1), except for the CCI. Patients in the long-duration group had a lower median CCI (1.0 vs. 5.5, p = 0.017) than those in the short-duration group. No differences were observed in the sources of bacteremia among the patients, and none were found to have multiple sources of infection. Additionally, none of the patients with pneumonia as the source of bacteremia received daptomycin monotherapy at any point during treatment.

Regarding infection management, less than half of the patients were determined to have source control measures (43.8%, 14/32), which was similar between the groups. Of the patients without source control measures, 66.7% (12/18) had a known focus requiring source control, and 33.3% (6/18) had no identifiable focus for source control. There was no statistically significant difference in endocarditis diagnosis between groups (p = 0.479). All the patients included in this study received an infectious disease consultation during their hospitalization.

In our cohort, eight patients died within 30 days of the onset of bacteremia: seven in the short-duration group and one in the long-duration group (p = 0.04). Of these patients, four died while receiving combination therapy, each having been on it for more than seven days. Additionally, two patients died within 24 hours after discontinuing combination therapy, while another death occurred three days after discontinuation, and a third six days after discontinuation. The cause of death in these patients was primarily related to complications from MRSA bacteremia, such as intracranial hemorrhage, cerebral herniation, or refractory septic shock. In univariate analysis, prolonging the duration of combination therapy after bacteremia clearance was associated with a lower risk of 30-day mortality (OR 0.09, 95% CI 0.004–0.59, p = 0.03). However, this association was no longer observed in the multiple logistic regression model after controlling for confounders such as CCI and PBS (AOR 0.17, 95% CI 0.007–1.85, p = 0.18) (Table 2).

**Table 2:** Multiple logistic regression analysis of 30-day all-cause mortality outcome.

Variable	Adjusted Odds Ratio (95% CI)	P value
**Intercept**	0.11 (0.006–0.88)	0.07
**≥ 7 days duration of combination therapy**	0.17 (0.007–1.85)	0.18
**CCI**	1.21 (0.88–1.77)	0.26
**PBS**	1.50 (1.00–2.49)	0.07

Abbreviations: CCI, Charlson comorbidity index; CI, confidence interval; PBS, Pitt bacteremia score.

Bacteremia recurrence was observed in two patients in the cohort, both of which were in the long-duration group (6.2%, p = 0.47). No significant association was observed between the duration of combination therapy after bacteremia clearance and bacteremia recurrence (OR 2.53, 95% CI 0.19–inf, p = 0.24).

The median post-bacteremia clearance length of stay among the 32 patients was 14.5 days (IQR 7.25, 29.25). Patients in the long-duration group had a longer median post-bacteremia clearance length of stay of 21 days (IQR 14.75, 30.5) compared to six days (IQR 5, 10.75) in the short-duration group (p = 0.002). After controlling for overall hospitalization, no difference was observed in the post-bacteremia clearance length of stay between the two groups (p = 0.37).

Four patients (12.5%) experienced ADE: one in the short-duration group and three in the long-duration group (p = 0.59), with AKI being the only ADE observed in our study. No significant association was observed between the duration of combination therapy after bacteremia clearance and ADE (OR 3.46, 95% CI 0.39–74.86, p = 0.31).

## Discussion

MRSA bacteremia is associated with significant attributable mortality and disease burden [2–4, 12]. Mortality rates for persistent bacteremia are significant and increase the longer the bacteremia persists. Studies have reported up to 45.2% mortality with bacteremia lasting longer than seven days, compared to 9.4% with bacteremia clearing in less than three days [13]. Prior studies have shown rapid bacteremia clearance and lower mortality rates when an anti-staphylococcal beta-lactam is used in combination with either vancomycin or daptomycin for the treatment of persistent MRSA bacteremia [14–18]. Additionally, ceftaroline has demonstrated efficacy as a monotherapy in the treatment of MRSA bacteremia, showing promising outcomes with regard to clinical and microbiological cure [19, 20]. However, its role in combination therapy and optimal duration of treatment remain unknown. This is a critical question that needs to be answered as its use becomes more prevalent.

While few studies have evaluated combination therapy with vancomycin or daptomycin alongside ceftaroline and reported mostly favorable outcomes and safety in this practice [15, 21, 22], our study (to the best of our knowledge) represents the first attempt to describe the impact of ceftaroline combination therapy duration following bacteremia clearance. The results of our study suggest that continuing ceftaroline combination therapy for prolonged periods (i.e., ≥ 7 days) beyond bacteremia clearance did not result in reduced 30-day all-cause mortality. This suggests that the duration of combination therapy may not play a significant role in reducing mortality in patients with persistent or high-grade MRSA bacteremia.

The mortality rate observed in our cohort (25%) is in line with previous reports, which described 30-day mortality rates due to MRSA bacteremia ranging from 13% to 39% [23–26]. While McCreary et al. concluded that early combination therapy may be beneficial in reducing mortality risk [27], our findings are consistent with those of previous studies that found no significant reduction in mortality when combination therapy was initiated later in the disease course [9, 16–18, 28–32]. In addition, it is worth noting that while we observed a significant difference in mortality between the long- and short-duration treatment groups in the univariate analysis, the short-duration group had a higher median CCI (5.5 vs 1.0) than the long-duration group, which may explain the observed differences in 30-day all-cause mortality. This is further supported by the fact that no significant association between 30-day mortality and the duration of combination therapy was observed after adjusting for CCI and PBS.

Furthermore, we did not find any association between the duration of combination therapy and bacteremia recurrence, length of hospital stay post-bacteremia clearance, or adverse drug events. These findings suggest that there may be no added benefit to continuing ceftaroline combination therapy for prolonged periods beyond the date of bacteremia clearance. Bacteremia recurrence was observed in two patients in the long-duration combination group. These patients did not have adequate source control measures, which is a likely reason for bacteremia relapse. As noted in previous studies, source control remains one of the cornerstones of MRSA bacteremia management [11, 30–32].

None of the patients experienced *C*. *difficile* colitis or neutropenia, but four patients experienced AKI; only one of them was in the short-duration group. Although there were no statistically significant differences between the two groups, possibly because of our small sample size, the CAMERA2 trial had similar findings that led to early termination owing to safety concerns in patients receiving combination therapy for prolonged durations of seven days or more [9]. A study by Pujol et al. also found a higher incidence of treatment-limiting ADE in the combination therapy group than in the monotherapy group, consistent with our findings [33].

The most common reason for prolonging combination therapy in our cohort was provider preference, which ranged from seven days to six weeks. Provider preferences may have been driven by concerns about the severity of illness and presence of comorbidities in patients, which led to a perceived need for extended treatment. However, the median PBS was higher in the short-duration group than in the long-duration group, indicating that the patients in the short-duration group may have had more severe illness at the time of bacteremia. As such, we believe that the clinical practice variations in our cohort likely stem from the lack of published guidance for the optimal duration of combination therapy once bacteremia clearance occurs.

The goal of combination therapy for persistent or high-grade MRSA bacteremia is to achieve rapid and sustained clearance, while minimizing potential adverse effects. Therefore, it is crucial to strike a balance between the duration of combination therapy and the risk of adverse drug events. Overall, while our study did not find a statistically significant reduction in mortality, bacteremia recurrence, adverse drug events, or post-bacteremia clearance length of stay with prolonged combination therapy, it is important to interpret these findings within a broader clinical context. The decision to discontinue ceftaroline should be made in consultation with an infectious diseases specialist, and should be guided by the patient’s individual clinical response. Considering the theoretical benefits, limiting the duration of combination therapy to the period of active bacteremia has the potential advantage of circumventing antimicrobial resistance, adverse drug events, and collateral damage in the form of *C*. *difficile* colitis. However, more research is needed to determine the optimal duration and role of combination therapy and to establish evidence-based guidelines for managing persistent MRSA bacteremia.

Although our study did not find a significant reduction in mortality, it is important to interpret these findings cautiously due to several limitations. First, this was a retrospective study with a relatively small sample size. While we employed multiple regression techniques to address confounding issues, the small sample size limited the precision of our estimates and, consequently, our ability to detect small effects. The lack of statistically significant findings may partly stem from limitations in statistical power. Additionally, the small sample size made it difficult to employ a matched study design, because there were insufficient patients to draw matched controls. Second, the single-center nature of this study limits its generalizability, and may not reflect the practices and outcomes of other healthcare settings. Third, the decision to continue ceftaroline after bacteremia resolution may have been influenced by factors outside of the variables collected in this study. The retrospective nature of this study and the lack of standardized documentation in the medical records also limited our ability to ascertain the precise reasons for the choice of prolonged combination therapy in some patients. Despite these limitations, our study contributes to the existing literature by providing important insights into the use of combination therapy for persistent or high-grade MRSA bacteremia. Further research is needed to determine the optimal duration and role of combination therapy for persistent or high-grade MRSA bacteremia.

## Conclusions

In this single-center retrospective study, prolonging the duration of ceftaroline combination therapy after bacteremia clearance did not result in a statistically significant reduction in mortality, bacteremia recurrence, adverse drug events, or post-bacteremia clearance length of stay in patients with persistent or high-grade MRSA bacteremia. However, it is important to interpret these findings with caution due to the limitations of the study design and sample size. Further research is needed to better understand the effectiveness of combination therapy for persistent or high-grade MRSA bacteremia, including larger randomized controlled trials that can provide more robust evidence.
